# Intraocular Amphiregulin antibody and axial elongation in nonhuman primates

**DOI:** 10.3389/fopht.2022.995157

**Published:** 2022-09-30

**Authors:** Wenyao Wang, Yan Nan, Tiejun Huang, Mingliang Pu, Jost B. Jonas

**Affiliations:** ^1^ Department of Anatomy/Embryology, School of Basic Medical Sciences, Peking University, Beijing, China; ^2^ Beijing Academy of Artificial Intelligence, Beijing, China; ^3^ Department of Computer Science and Technology, School of Electronics Engineering and Computer Science, Peking University, Beijing, China; ^4^ Department of Ophthalmology, Medical Faculty Mannheim, University of Heidelberg, Mannheim, Germany; ^5^ Institute of Molecular and Clinical Ophthalmology Basel, Basel, Switzerland

**Keywords:** epidermal growth factor, amphiregulin, myopia, axial elongation, myopic maculopathy

## Abstract

**Purpose:**

To examine the effect of intraocularly applied amphiregulin antibody on physiological axial elongation in young nonhuman primates.

**Methods:**

The experimental study included six male 12-months-old macaque nonhuman primates (body weight:2.46 ± 0.25kg;range:2.20-2.90kg). In the experimental group (n=3 animals), three intravitreal injections of amphiregulin antibody (100μg/50μl) were applied to the left eyes at intervals of 4-6 weeks, and injections of phosphate buffered solution (50μl) were applied to the right eyes. Three other animals were assigned to a blank control group.

**Results:**

During the study period of 23.6 weeks, axial length in the experimental group did not change in the left eyes (18.91 ± 0.37mm to 18.94 ± 0.67mm;*P*=0.90), while it linearly increased in the right eyes (18.87 ± 0.38mm to 19.24 ± 0.53mm;*P*=0.056) and in the control group (left eyes:19.15 ± 0.22mm to 19.48 ± 0.22mm;*P*=0.009; right eyes:19.17 ± 0.15 mm to 19.46 ± 0.23 mm;*P*=0.024). The interocular difference in axial elongation increased in the experimental group from -0.11 ± 0.12mm at 4 weeks after baseline to -0.34 ± 0.15mm at the study end, while in the control group, the interocular side difference did not change significantly (from 0.01 ± 0.10 mm to 0.03 ± 0.08 mm;*P*=0.38). The difference in the interocular difference in axial elongation between the two groups was significant at 8 weeks (*P*=0.01), 15 weeks (*P*=0.007), and at study end (*P*=0.02). The interocular difference in axial length correlated with the interocular difference in vitreous cavity length (standardized regression coefficient beta:0.85;*P*<0.001). The interocular axial length difference was inversely associated with the interocular refractive error difference (beta:-0.49;*P*<0.001).

**Conclusions:**

Intraocularly applied amphiregulin antibody (100μg) reduced the physiological ocular axial elongation in juvenile nonhuman primates.

## Introduction

Axial myopia is one of the most common causes of irreversible vision impairment and blindness worldwide. It is characterized by a sagittal eye diameter being too long in relationship to the combined refractive power of the cornea and lens (1). It has been discussed that axial myopia is the result of an overshooting of the physiological process of emmetropization. The process of emmetropization describes the physiological axial elongation of the eye by which the marked axial hyperopia present at birth is transformed into emmetropia in adulthood. For each millimeter of the ocular axial length getting too long in the relationship to the refractive power of cornea and lens, a myopic refractive error of approximately -3 diopters results. Previous studies have suggested that axial elongation stops in the third decennium in moderately myopic individuals (i.e., refractive error of less than -6 to -8 diopters or axial length of less than 26.0 mm to 26.5 mm). In contrast, highly myopic patients, in particular those with pathologic changes in the macula (i.e., myopic maculopathy) or the optic nerve (high myopia-associated optic neuropathy), can experience further axial elongation in adulthood so that sagittal eye diameters of more than 35 mm (normal: 23.6 mm) can eventually result (2–6). Continuous axial elongation is a main risk factor for the progression of myopic maculopathy (3–7). Procedures are therefore warranted to decrease or completely stop further axial elongation and myopization in highly myopic eyes to prevent the development and progression of myopic maculopathy and high myopia-associated optic neuropathy.

Previous experimental and clinical studies have reported that various molecules are potentially involved in the process of axial elongation. These molecules include dopamine, atropine, transforming growth factor beta, fibroblast growth factor, hepatocyte growth factor, insulin-like growth factor, and amphiregulin and other epidermal growth factor (EGF) family members (8–21). Recent studies conducted in young guinea pigs with or without lens-induced myopization showed that the repeated intravitreal application of antibodies against EGF family members, such as amphiregulin, neuregulin-1 and EGF itself, resulted in a decrease in axial elongation, while repeated intravitreal injections of EGF family members themselves, namely, amphiregulin, neuregulin-1 and EGF, were associated with an increase in axial elongation (18–21). As a corollary, the intravitreal application of EGF receptor antibodies was associated with a reduction in the axial elongation in young guinea pigs (21). The hypothesis was formulated that EGF and its family members, potentially produced by cells in the retina, stimulate the retinal pigment epithelium (RPE) in the midperiphery of the fundus to locally enlarge Bruch´s membrane (BM) as its basal membrane. The BM enlargement in the midperiphery would elongate the eye and change the eye shape from a sphere to a prolate form (22). Histomorphometric studies on human globes have supported the notion of BM as a biomechanically important structure for the shape and size of the eye (23–29). These investigations revealed that BM thickness was independent of axial length, so that BM volume increased with a longer axial length; that the volume of the sclera and choroid, in contrast to BM volume, was independent of axial length; that the retinal thickness and the RPE cell layer density in the macula were not related to the axial length, while both parameters in the fundus periphery decreased with a longer axial length; and that the axial elongation-associated increase in the optic disc-fovea distance was due to the development and enlargement of parapapillary, BM-free, gamma zone, while the length of BM in the macular region was independent of axial length. Based on these histomorphometric findings and intravital observations made in guinea pigs (18–21), we examined whether an intraocularly applied antibody against amphiregulin as an EGF family member influences the ocular axial elongation in nonhuman primates.

## Methods

The experimental study included six male juvenile macaque nonhuman primates (*Macaca fascicularis*) aged 11 months. The animals were purchased from a regional primate center (Beijing Xie Er Xin Biology Resource Co., Ltd., Beijing, China). All animal experiments and procedures were approved by the Institutional Animal Care and Use Committee IACUC of Peking University (No. LA2017276) and were performed in accordance with the Association for Research in Vision and Ophthalmology (ARVO) statement for the use of animals in ophthalmic and vision research. The reporting in this manuscript follows the recommendations outlined in the ARRIVE (Animal Research: Reporting of *In Vivo* Experiments) guidelines. The nonhuman primates were kept in a 12 h/12 h light and dark environment with a constant room temperature of 26°C. Food and water were provided ad libitum. Each animal in the experimental group received three injections with intervals of 4-6 weeks between the injections. The reason for choosing an interval of 4-6 weeks between the injections was that in previous studies using guinea pigs, the injections were performed at similar intervals of 4 weeks (18–20). The animals in the control group did not undergo any procedures. The study duration was five months.

The three nonhuman primates of the experimental group received intravitreal injections of 100 μg human amphiregulin antibody (AF262, R&D Systems, Inc. Minneapolis, MN, USA) in 50 μL of 0.1 M phosphate buffered solution (PBS) (HLX-P10015, Double-Helix Biotech, Shanghai, China) in their left eyes and received intravitreal injections of 50 μL of 0.1 M PBS into their right eyes. The dose of 100 μg human amphiregulin antibody that was injected into the nonhuman primates with an axial length of approximately 20 mm or an ocular volume of 3.6 mL corresponded to doses of 5 μg, 10 μg and 15 μg amphiregulin antibody injected into the eyes of guinea pigs with an axial length of 8 mm and an ocular volume of 0.27 mL in previous studies (18–20).

**Figure 1:**
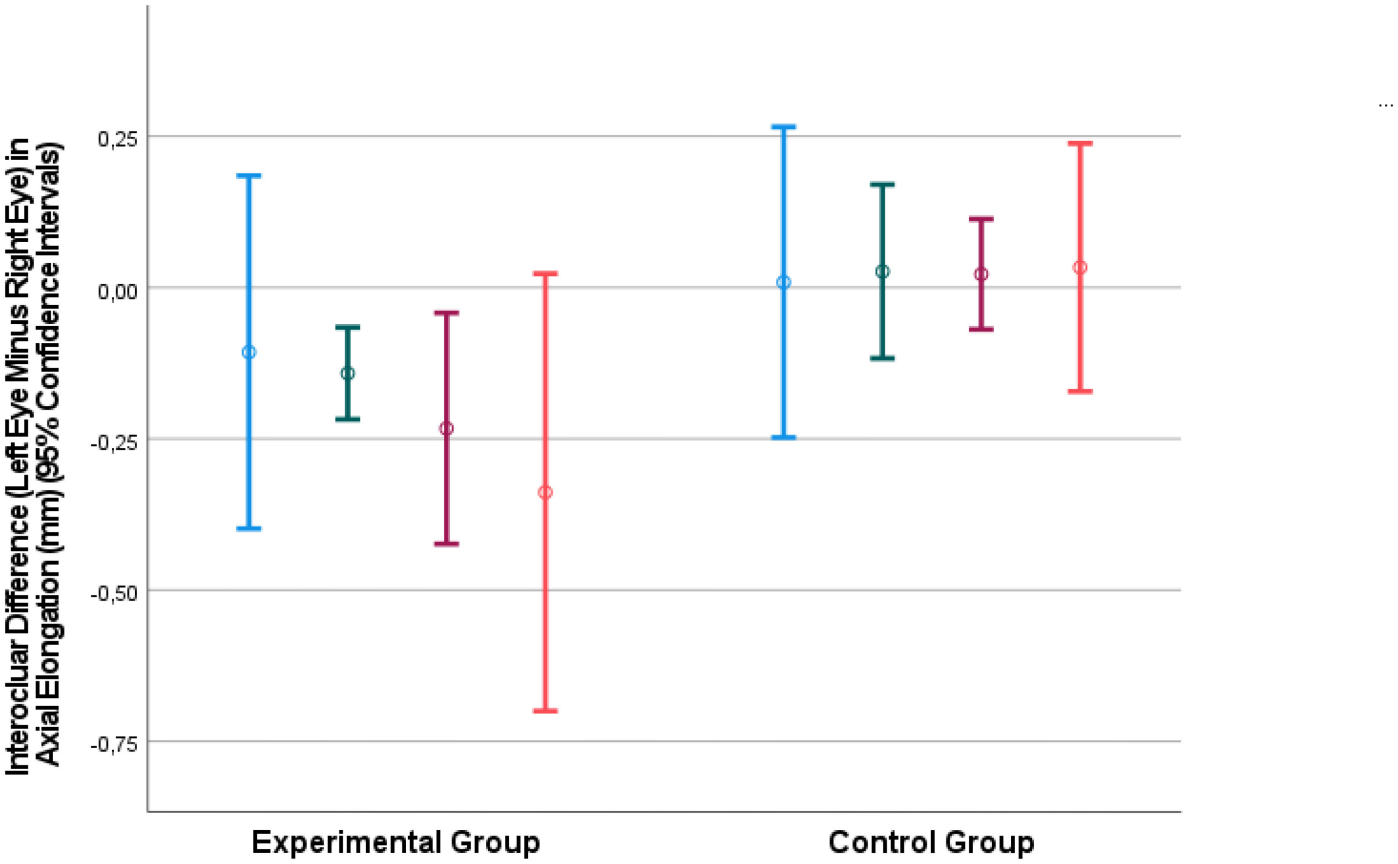
Interocular difference in the axial elongation (mm) (left eye minus right eye) at 4 weeks after baseline (blue bars), 8 weeks after baseline (green bars), 14.6 weeks after baseline (fed bars) and 23.6 weeks after baseline (ornage bars).

All nonhuman primates underwent ophthalmological examinations under general anesthesia induced by an intramuscular injection of ketamine (1 mg/100 g; Gutian Pharmaceutical Co, Ltd, Fujian, China) (**Figure 1**). These examinations were performed three times within four weeks before the first injection was given. They were repeated at weekly intervals after the start of the study during the whole study period until the end of the study. The examinations included the inspection of the external eye, an assessment for any signs of any external or internal inflammation, tonometry (Tono-pen AVIA, Reichert, Inc., Depew, NY, USA), and sonographic biometry (Axis nano, Quantel Medical, Cournon, French) for measurement of the axial length, anterior chamber depth, lens thickness and vitreous cavity length. The sonography was performed five minutes after topical application of oxybuprocaine eye drops (Santen Pharmaceutical Co., Ltd, Osaka, Japan); compound chondroitin sulfate eye drops (Mentholatum Pharmaceutical Co., Ltd, Guangzhou, China) were used to keep the cornea moist. For each time point, we performed three biometric measurements, with each measurement consisting of 10 readings. The average of all of the measurements was taken for further statistical analysis. After medical induction of pupillary mydriasis (tropicamide and phenylephrine eye drops; Santen Pharmaceutical Co., Ltd, Osaka, Japan), a direct ophthalmoscopic examination of the fundus (3.5 V Coaxial Ophthalmoscope, Welch Allyn, Inc. Milwaukee, WI) and a retinoscopy (HPX Streak Retinoscope, Welch Allyn, Inc. Milwaukee, WI) for measurement of the refractive error was performed.

Before the intravitreal injections were carried out, eye drops containing 5% iodine solution (Nanjing Nanda Pharmaceutical Industry Co., Ltd., Nanjing, China) were applied as a preoperative topical antiseptic. Using insulin syringes (Shurui, 328421, Becton Dickinson and Company, NJ), intravitreal injections were performed at 3 mm posterior to the temporal superior limbus, with the tip of the needle directed toward the posterior pole. After the injections, antibiotic eye drops containing ofloxacin (Renhe Pharmacy Co, Nanchang, Jiangxi, China) were applied.

The statistical analysis was conducted using a commercially available statistical software (SPSS for Windows, version 27.0, IBM-SPSS, Chicago, IL, USA). The normal distribution of the parameters was tested by applying the Kolmogorov–Smirnov test and the Shapiro–Wilk test. We assessed the mean ± standard deviation of the main outcome parameter, i.e., the axial length. The statistical significance of the differences between the study eye and the control eye and between different time points were determined by performing Student´s t-test for paired samples. The measurements between the groups were compared by applying Student´s t-test for unpaired samples. A multivariable linear regression analysis assessed the associations between the interocular difference in axial elongation and other ocular parameters. We calculated the standardized regression coefficient beta, the non-standardized regression coefficient B and its 95% confidence intervals (CIs). All *P*-values were two-sided and were considered statistically significant when the values were less than 0.05.

## Results

The animals were 12 months old at the time of the first injection. The mean body weight at that time point was 2.46 ± 0.25 kg (range: 2.20–2.90 kg). Mean axial length at baseline was 19.01 ± 0.31 mm in the right eyes and 19.03 ± 0.30 mm in the left eyes, without significant difference (*P*=0.68). At baseline, the eyes of the experimental group and those of the control group did not significantly differ in axial length (right eyes: 18.87 ± 0.38 versus 19.17 ± 0.15 mm; *P*=0.31; left eyes: 18.91 ± 0.37 mm versus 19.15 ± 0.22 mm; *P*=0.40). Intraocular pressure (IOP) at baseline was 9.9 ± 0.7 mm Hg in the right eyes and 9.4 ± 1.3 mm Hg in the left eyes, without a significant difference between both eyes (*P*=0.28).

During the study period, the axial length increased in the blank control group from 19.17 ± 0.15 mm to 19.46 ± 0.23 mm in the right eyes and from 19.15 ± 0.22 mm to 19.48 ± 0.22 mm in the left eyes (**Table 1**). In the experimental group, only the right eyes showed a linear axial elongation, from 18.87 ± 0.38 mm at baseline to 19.24 ± 0.53 mm at the end of the study. In the left eyes, axial length did not significantly change from baseline to the end of the study (18.91 ± 0.37 mm to 18.94 ± 0.67 mm; *P*=0.90).

**Table 1:** The axial length in nonhuman primates (mean ± standard deviation).

Group	n	Eye	Baseline (age: 11 months)	First injection (age: 12 months) (baseline + 4 weeks)	Second injection (age: 13 months) (baseline + 8 weeks)	Third injection (age: 14.4 months) (baseline + 14.6 weeks)	Follow-up (age: 16.5 months) (baseline + 23.6 weeks)
Axial length (mm)							
Experimental Group	3	Right	18.87 ± 0.38	18.98 ± 0.43	19.07 ± 0.52	19.10 ± 0.56	19.24 ± 0.53
		Left	18.91 ± 0.37	18.91 ± 0.36	18.97 ± 0.55	18.90 ± 0.49	18.94 ± 0.67
*P-*value (difference right/left eye)			0.41	0.27	0.10	0.08	0.08
Control Group	3	Right	19.17 ± 0.15	19.25 ± 0.23	19.26 ± 0.19	19.39 ± 0.23	19.46 ± 0.23
		Left	19.15 ± 0.22	19.25 ± 0.20	19.27 ± 0.21	19.39 ± 0.26	19.48 ± 0.22
*P*-value (difference right/left eye)			0.75	0.84	0.24	0.73	0.14
Interocular difference in the axial length (left eye minus right eye) (mm)							
Experimental Group	3		-0.04 ± 0.06	-0.07 ± 0.08	-0.10 ± 0.06	-0.19 ± 0.10	-0.30 ± 0.16
Control Group	3		-0.01 ± 0.07	-0.01 ± 0.04	0.01 ± 0.01	0.01 ± 0.03	0.02 ± 0.01
*P*-value (difference between both groups)			0.13	0.27	0.05	0.05	0.046
Interocular difference in the axial elongation							
Experimental Group	3			-0.11 ± 0.12	-0.14 ± 0.03	-0.23 ± 0.08	-0.34 ± 0.15
Control Group	3			0.01 ± 0.10	0.03 ± 0.06	0.02 ± 0.04	0.03 ± 0.08
*P value*				0.27	0.01	0.007	0.02

In the blank control group, the interocular side difference in axial length (left eye minus right eye) was close to zero during the whole study period and did not change significantly (**Table 1**). In the experimental group, the interocular difference in axial length (left eye minus right eye) increased from -0.04 ± 0.06 mm to -0.30 ± 0.16 mm (**Table 1**). The difference between the two groups in the interocular difference in the axial length was significant for the values measured at the end of the study (*P*=0.046) (**Table 1**).

The interocular difference in axial elongation increased in the experimental group from -0.11 ± 0.12 mm at 4 weeks after baseline to -0.34 ± 0.15 mm at the end of the study (**Table 1**). The difference between the two groups in the interocular difference in axial elongation was significant at 8 weeks (*P*=0.01), 15 weeks (*P*=0.007), and at the end of the study (*P*=0.02) (**Table 1**) (**Figure 1**).

**Figure 2:**
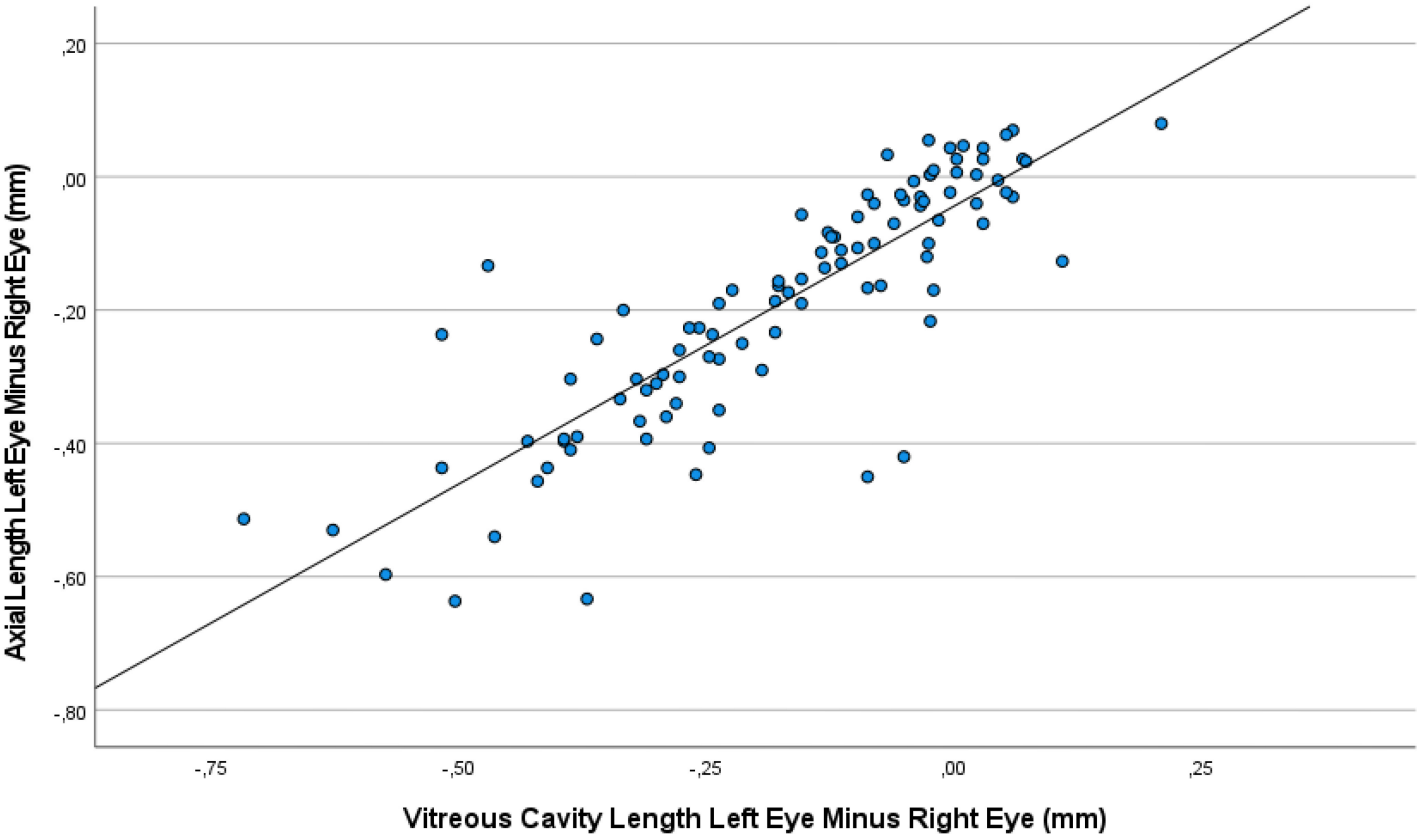
Graph showing the association between the interocular difference in the vitreous cavity length and the interocular difference in the axial length, with the measurements taken at baseline and at various examination during the follow-up period.

The interocular difference in axial length was strongly correlated with the interocular difference in the vitreous cavity length (Interocular Axial Length Difference = 0.83 x Interocular Vitreous Cavity Length Difference -0.04; beta: 0.85; 95% CI for B: 0.73, 0.94; *P*<0.001) (**Figure 2**). The association between the interocular difference in axial length and the interocular difference in anterior chamber depth was less significant (Interocular Axial Length Difference = 0.79 x Interocular Anterior Chamber Depth Difference -0.17; beta: 0.50; 95% CI for B: 0.52, 1.07; *P*<0.001).

**Figure 3:**
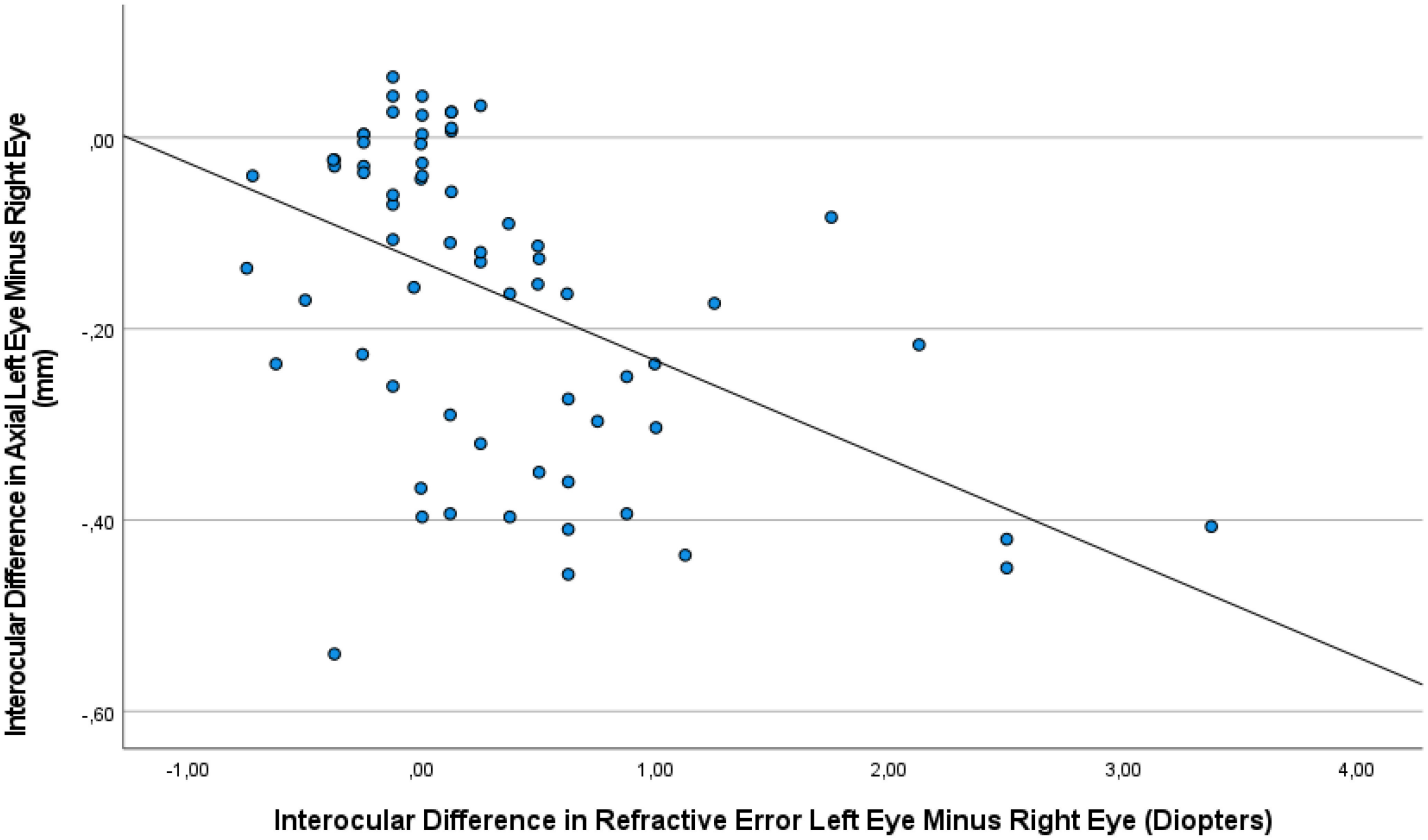
Graph showing the association between the interocular difference in refractive error and the interocular difference in axial length, with the measurements taken at baseline and at various examination during the follow-up period.

The interocular difference in axial length was correlated with the interocular difference in refractive error (Interocular Axial Length Difference = -0.10 x Interocular Refractive Error Difference -0.13; beta: -0.49; 95% CI for B: -0.15, -0.06; *P*<0.001) (**Figure 3**). No signs of toxicity or injection-related changes in the retina or any other intraocular tissue were observed in any of the animals in the study.

## Discussion

The results of our experimental study showed that repeated intravitreal applications of amphiregulin antibody at a dose of 100 μg was associated with a significant decrease in axial elongation in young nonhuman primates (**Figure 1**). This finding was in agreement with the notion that epidermal growth factor family members may be involved in the process of axial elongation.

Since other studies have not yet examined the influence of an intraocularly applied antibody against an EGF family member on the physiological axial elongation in nonhuman primates, the results of our study cannot be directly compared with observations made in other investigations. Our study results agree with the findings obtained in previous investigations on guinea pigs in which the intravitreal application of antibodies against EGF, EGF receptor and EGF family members amphiregulin, neureglin-1, epiregulin, epigen and betacellulin resulted in a decrease in axial elongation (18–21). The observations were made in animals with lens-induced myopization and in animals with physiological eye growth without any intervention to induce myopization (18–21). As a corollary, guinea pigs that received intravitreal injections of amphiregulin (20 μg) and neuregulin-1 (0.05 μg, 0.10 μg, and 0.20 μg) showed a dose-dependent increase in the axial elongation (19). The results of our study extend the results of the previous investigations on guinea pigs into the field of nonhuman primates.

The hypothesis that EGF, including its family members, is associated with the process of axial elongation has been based on clinical and histomorphometric studies, namely, that an enlargement of BM in the midperipheral region may lead to a backward push of the posterior part of BM, with secondary compression and thinning of the choroid and evasion of the posterior sclera. Such a mechanism could explain choroidal thinning, which cannot be explained if the sclera is assumed to be the tissue that primarily elongates the eye (22). If the sclera were the tissue elongating the eye, the distance between the sclera and BM, i.e., the choroidal thickness, would become wider. The BM-based mechanism of axial elongation fits with the observation that the axial elongation-associated thinning of the choroid is, in absolute and relative terms, more marked than the thinning of the sclera. It also agrees with the observation that the best corrected visual acuity in eyes without myopic maculopathy is independent of axial length, since a BM enlargement in the fundus midperiphery does decrease the density of the retinal photoreceptors in the macular region. Correspondingly, studies have shown that the increase in the disc-fovea distance in axially elongating eyes is due to the development of the BM-free parapapillary gamma zone, while the length of BM in the macular region in the horizontal and vertical directions was unaffected by axial elongation (22, 27, 30). Correspondingly, histomorphometric investigations revealed that the thickness and density of the choriocapillaris and RPE in the macular region and BM thickness were independent of axial length (25–28).

The findings of our investigation may be of clinical importance. Recent studies on highly myopic adult patients revealed that highly myopic eyes can further elongate even at the age of 50+ years and that such axial elongation is a major risk factor for the progression of myopic maculopathy (31). If the hypothesis of EGF as an axial elongation-associated molecule is valid and if the results of our study on nonhuman primates can be transferred to patients, it may imply, that an antibody against EGF, an EGF family member or the EGF receptor could be considered to prevent further axial elongation in highly myopic adult patients with continuing myopic eye enlargement. Interestingly, EGF receptor antibodies, such panitumumab, have been clinically used for decades in oncology as therapy against some forms of colon carcinoma and squamous cell carcinoma of the head and neck (32). Clinical studies on the systemic use of EGF receptor antibodies did not reveal an intraocular toxicity. Correspondingly, we did not observe toxic effects of the intraocularly applied amphiregulin antibody in our study. This finding fits with the observations made in guinea pigs, in which intravital assessment and histomorphometric and immunohistochemical examinations of enucleated eyes did not show signs of tissue loss or inflammation after the intraocular application of antibodies against EGF, its family members, and the EGF receptor (18–21).

When the results of our study are discussed, its limitations must be taken into account. First, the number of animals in our study was small, so the statistical power of the study was low. Despite of this limitation, the differences in the interocular difference in axial elongation between the study group and control group were statistically significant (**Table 1**) (**Figure 1**). Second, the nonhuman primates did not undergo lens-induced or form-deprivation myopization, so that we did not examine the influence of the intravitreally applied amphiregulin antibody on externally induced axial elongation, nor did we explore the effect of intravitreal amphiregulin antibody on the natural progression of myopia. Third, the unilateral injection of amphiregulin antibody might have affected the axial elongation of the contralateral eye. If, however, the contralateral (control) eyes would have been inhibited in their growth, the inter-eye difference would have decreased. This potential limitation of our study might thus only serve to strengthen the findings of our study. Fourth, we did not investigate whether the administered antibody showed binding within the eye or whether it induced its expected EGF receptor effects, so the results could also have been driven by off-target or immune-based effects. Fifth, we did not perform an optical coherence tomographic examination of the retina, so that our observation of an intraocular compatibility of the intravitreally applied amphiregulin antibody was based on ophthalmoscopic and slit-lamp-based examinations. Sixth, we included *Macaca fascicularis* nonhuman primates in this study, while previous studies often included rhesus nonhuman primates. Future investigations may address whether a difference between nonhuman primate species may influence the effect of intraocularly applied amphiregulin antibody on the axial elongation and its generalizability to humans. Seventh, we did not examine toxicological effects of the intraocularly injected amphiregulin antibody, so that future studies may explore potential histological and immunohistochemical changes induced by the intravitreally applied amphiregulin antibody. Eighth, the experimental procedures started when the primates were at an age of 12 months, but nonhuman primates complete the early phase of their ocular growth and emmetropization by the age of 5 months. The results of our study may thus, however, suggest that the amphiregulin antibody had an effect beyond the most active phase of physiological axial elongation in nonhuman primates. Due to the coronavirus pandemic, an earlier start for the study was not possible. One may discuss that if the amphiregulin antibody was effective in the relatively old nonhuman primates (with an age of 12 months), it might be even more effective if it had been applied in younger nonhuman primates.

In conclusion, the repeated intravitreal injection of 100 μg amphiregulin antibody was associated with a significant and cumulative reduction in the physiological axial elongation in nonhuman primates aged 12 months. The results agree with the notion that the EGF system is associated with the process of axial elongation in nonhuman primates.

## Data availability statement

The raw data supporting the conclusions of this article will be made available by the authors, without undue reservation.

## Ethics statement

The animal study was reviewed and approved by All animal experiments and procedures were approved by the Institutional Animal Care and Use Committee IACUC of Peking University (No. LA2017276) and were performed in accordance with the Association for Research in Vision and Ophthalmology (ARVO) statement for the use of animals in ophthalmic and vision research.

## Author contributions

Design: WW, YN, TH, MP, JJ. Measurements: WW, YN, TH, MP. Supervision: MP. Funding: MP, JJ. Statistical analysis: WW, YN, TH, MP, JJ. First draft of the manuscript: WW, YN, TH, MP, JJ. Revising and approval of the final version of the manuscript: WW, YN, TH, MP, JJ. All authors contributed to the article and approved the submitted version.

## Funding

This work was supported by the National Science Foundation of China (31571091 to MP), National Basic Research Program of China (2015CB351806 to MP), National Science Foundation of China (61425025 to TH).

## Conflict of interest

JJ holds European patent EP 3 271 392, JP 2021-119187, and US 11,008,385: “Agents for use in the therapeutic or prophylactic treatment of myopia or hyperopia, and has Patent application: European patent application: WO 2021/198369 A1; PCT/EP2021/058500: Agents for use in the therapeutic or prophylactic treatment of retinal pigment epithelium-associated diseases.

The remaining authors declare that the research was conducted in the absence of any commercial or financial relationships that could be construed as a potential conflict of interest.

## Publisher’s note

All claims expressed in this article are solely those of the authors and do not necessarily represent those of their affiliated organizations, or those of the publisher, the editors and the reviewers. Any product that may be evaluated in this article, or claim that may be made by its manufacturer, is not guaranteed or endorsed by the publisher.
